# Manipulating antigen presentation for antigen-specific immunotherapy of autoimmune diseases

**DOI:** 10.1016/j.coi.2021.03.019

**Published:** 2021-06

**Authors:** Heather B Streeter, David C Wraith

**Affiliations:** Institute of Immunology and Immunotherapy, University of Birmingham, B15 2TT, United Kingdom

## Abstract

Current treatments for autoimmune diseases do not address the immune pathology underlying their initiation and progression and too often rely on non-specific immunosuppressive drugs for control of symptoms. Antigen-specific immunotherapy aims to induce tolerance selectively among the cells causing the disease while leaving the rest of the adaptive immune system capable of protecting against infectious diseases and cancers. Here we describe how novel approaches for antigen-specific immunotherapy are designed to manipulate antigen presentation and promote tolerance to specific self-antigens. This analysis points to liver antigen presenting cells, targeted by carrier particles, and steady-state dendritic cells, to which antigen-processing independent T-cell epitopes (apitopes) bind directly, as the principal targets for antigen-specific immunotherapy. Delivery of antigens to these cells holds great promise for effective control of this rapidly expanding group of diseases.

## Highlights

• Specific immunotherapy is the ‘holy grail’ for treatment of autoimmunity.
• Antigens are delivered by either direct or indirect presentation mechanisms.
• Liver APC and steady state DC mediate distinct forms of immune regulation.
• Tr1 cell induction involves epigenetic modification of tolerance associated genes.
• Trials reveal that antigen-specific immunotherapy can control autoimmune diseases.

**Current Opinion in Immunology** 2021, **70**:75–81

This review comes from a themed issue on **Antigen processing**

Edited by **Scheherazade Sadegh-Nasseri** and **Sebastian Springer**

For a complete overview see the Issue and the Editorial

Available online 18th April 2021

**https://doi.org/10.1016/j.coi.2021.03.019**

0952-7915/© 2021 University of Birmingham, U.K. Published by Elsevier Ltd. This is an open access article under the CC BY license (http://creativecommons.org/licenses/by/4.0/).

## Introduction

Autoimmune diseases arise from immune responses to self-antigens and reflect a breakdown in immunological tolerance. Polymorphisms linked to autoimmune diseases map to genes regulating the adaptive immune system and their dysregulation leads to ineffective control of autoreactive T-cells and associated production of autoantibodies [1]. Most classical autoimmune diseases have associations with genes in the major histocompatibility complex (MHC) class II region and are often more common in women than men [2]. Recent years have seen a dramatic increase in the incidence and prevalence of autoimmune diseases [3] with an alarming increase in type I diabetes (T1D) amongst children of 0–4-years [4].

Current treatments for autoimmune diseases depend on replacement therapies or the continued use of non-specific immunosuppressive drugs. Immunosuppressive drugs fail to resolve the immune pathology underlying autoimmune diseases and reduce the ability of the treated individual to combat infections and cancers. An alternative strategy is antigen-specific immunotherapy (AIT) designed to reinstate immunological tolerance to self-antigen/s while leaving the immune system to function effectively.

AIT has been practiced by allergists for over a century. Noon and Freeman described how inoculation with ‘pollen toxin’ led to ‘a distinct amelioration of symptoms’ in affected individuals [5]. The ‘pollen toxin’ was an aqueous extract of pollen and suppression of disease by repeated injection laid the foundations for the allergic desensitization practiced today. Incremental improvements in desensitization protocols have included allergen purification; however, desensitization with whole allergen retains the risk of immune activation through cross-linking of mast cell bound IgE. This hazard is avoided by targeting allergen-specific CD4^+^ T-cells with T-cell epitopes since CD4^+^ cells orchestrate the allergic response [6]. Various attempts have been made to use intact proteins for treatment of autoimmune diseases. Oral tolerance induction proved successful in experimental models of autoimmune disease but did not translate to the clinic [7], presumably because an effective dose could not be reached [8]. Further attempts to treat autoimmune conditions with intact antigen even led to exacerbation of disease in models of MS [9], T1D [10] and Graves’ disease [11]. These examples strengthen the case for using T-cell epitopes for AIT of autoimmune diseases [12].

In developing AIT, several studies have designed altered peptide ligands (APL) based on the T-cell epitope in order to modulate interactions with either MHC or the T-cell receptor (TCR). Our laboratory has designed analogues of Ac1-9, the N-terminal epitope of myelin basic protein (MBP) that induces experimental autoimmune encephalomyelitis (EAE) in H-2^u^ mice. A heteroclitic MHC-binding analogue was shown to suppress disease by activation induced cell death in Ac1-9 specific T-cells [13]. APL were designed as TCR antagonists by altering two TCR interaction residues in Ac1-9 [14]. These were shown to antagonise activation of Ac1-9 specific T-cell clones *in vitro*. However, it was noted that an APL that served as an antagonist *in vitro* would induce EAE *in vivo* demonstrating that one peptide could be an antagonist for clone A but an agonist for clone B. Subsequent clinical trials of immunotherapy with an APL from MBP:83-99 led to disease exacerbation in an individual with MS [15]. In parallel trials of the APL, patients developed urticaria proving that this peptide was indeed immunogenic when injected subcutaneously at high dose [16]. These studies warn against the use of APL for AIT and raise important questions concerning the dose and solubility of the peptide that caused urticaria: subsequent studies have shown that soluble, native epitopes are well tolerated and effective for AIT of allergic [6,17^•^] or autoimmune diseases [18^••^,19^••^].

## Rationale for AIT in autoimmune diseases

**Figure 1:**
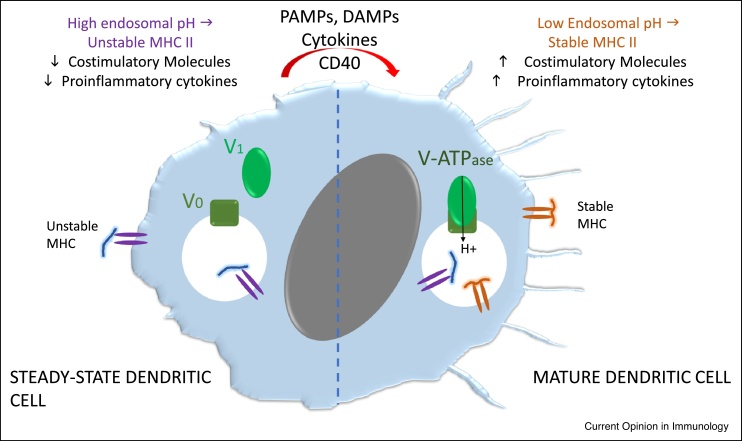
Properties of steady-state dendritic cells: ssDC *in situ* are relatively immature. They have low levels of both class II MHC and costimulatory molecules and secrete cytokines at low levels. ssDC undergo maturation under the influence of pathogen-associated molecular patterns (PAMPS), damage-associated molecular patterns (DAMPS), inflammatory cytokines such as TNF-α or CD40 ligation. The endosomal compartment in ssDC has a relatively high pH because of a low level of V-ATPase formed from its two subunits V_0_ and V_1_. The high pH in this compartment results in surface expression of unstable/peptide-receptive MHC II molecules. Endosomal acidification following formation of the V-ATPase in mature DC results in optimal function of the antigen presenting machinery stable MHC which with increased expression of costimulatory molecules results in T cell activation and differentiation into effector cells.

The immune response to self-antigens is controlled by both central (thymic) and peripheral tolerance. The properties of dendritic cells (DC) (Figure 1) and their role in peripheral tolerance are instructive. First, elimination of ‘steady-state’ DC (ssDC) in mice provokes uncontrolled lymphoproliferation and autoimmunity indicating that CD11c^+^ ssDC maintain peripheral tolerance [20,21]. Second, targeting antigen to ssDC using antibodies against surface receptors such as DEC-205 induced tolerance characterised by transient T-cell activation followed by cell death, anergy and the generation of regulatory T (Treg) cells [22,23]. The key point here is that this approach kept the targeted DC in the steady-state since no DC-activating inflammatory signals were provided.

## Novel approaches for AIT in autoimmune diseases

Current approaches being developed for AIT in autoimmune diseases include those targeting ssDC or directing antigens to liver APC (Table 1).

**Table 1:** Novel approaches in development for AIT of autoimmune diseases

Company	Delivery approach	Proposed mechanism of action	Impact on T cell response	Efficacy in experimental models	Clinical trial progress
Anokion	Antigens modified with polymeric forms of either *N*-acetylgalactosamine or *N-*acetylglucosamine	Target hepatic antigen-presenting cells	Induce CD4^+^ and CD8^+^ T-cell deletion and anergy	EAE^b^, T1D	Enrolling patients for KAN-101 trial in coeliac disease
Apitope International NV	Synthetic peptides designed as antigen processing independent CD4^+^ T cell epitopes (apitopes) injected in saline i.d. or s.c.	Bind selectively to steady-state DC in lymphoid organs	Induction of anergy and generation of regulatory T cells (Tr1 and Foxp3)	EAE and Graves’ disease models	Completed trials in MS and Graves’ disease
					Phase Ia in SPMS
					Phase Ib in RRMS
					Phase II in RRMS
					Phase I in Graves’ disease
Cellerys	Red blood cells (RBC) coupled with peptides from myelin in MS	Cell target macrophages and Kupffer cells in spleen and liver.	Increase in Tr1 cell response to antigen with reduced IFN-γ		Phase 1 in RRMS^c^
Cour/Takeda	Antigen encapsulated in PLG (poly(lactide-co-glycolide)) nanoparticles	Ag-PLG internalized by splenic marginal zone macrophages and liver phagocytic cells via scavenger receptors (MARCO)	Increase in Foxp3 Treg cells, dependent on CTLA-4, PD-1 and IL-10	EAE, T1D and Coeliac disease models	Phase I in patients with coeliac disease^e^
Dendright/Janssen Biotech Inc	Antigen with calcitriol in liposomes	Liposomes (105−135 nm) target steady-state DC in draining lymph nodes	Increase in Foxp3 Treg cells	Autoimmune arthritis and experimental Goodpasture’s vasculitis	Phase I in ACPA + rheumatoid arthritis^a^
Imcyse	T cell epitopes modified by addition of a thioredox motif (CXXC), injected in alum adjuvant	Promotes cytotoxic activity in T cells through increasing expression of granzyme B and FasL	Cytotoxic cells delete B cells in cognate recognition	T1D	Phase I in T1D recruiting for phase II^f^
Novo Nordisk	Plasmid DNA encoding proinsulin and co-expressing IL-10 and TGF-β	Promotes Treg cells	Promotes Treg cell differentiation	T1D with vector expressing GAD antigen	
Parvus	Nanoparticles coated with MHC II proteins and antigenic peptides	Bind directly to CD4^+^ effector cells	Drives differentiation of Tr1 cells from Th1 precursors in mice	EAE, CIA, T1D and autoimmune liver diseases	Phase I in coeliac disease planned for 2023
Selecta	PLG nanoparticles containing rapamycin co-administered with antigen	Nanoparticles found in dendritic cells in spleen and LSEC and Kupffer cells in the liver where they mediate down-regulation of CD80, 86, class II MHC and upregulation of PDL-1	Promotes Treg cell differentiation	EAE and anti-drug antibodies	Phase II in gout designed to block the anti-drug antibody response to pegadricase^d^
Tolerion	DNA encoding self-antigen	CpG islands in DNA replaced with GpG to reduce immunogenicity of antigen delivery	Promote immune regulatory response to self-antigen	BHT-3021 prevents T1D in mouse model	Phase I trial in T1D completed and phase II enrolling
Topaz	Ferromagnetic nanoparticles coupled to T cell epitopes	Nanoparticle-based autoantigen delivery to liver sinusoidal endothelial cells	Induction of Foxp3^+^ Treg cells in the liver	EAE	Phase I trial of TPM203 in pemphigus vulgaris

a https://acrabstracts.org/abstract/a-phase-i-randomized-double-blind-placebo-controlled-single-center-single-dose-escalation-to-investigate-the-safety-tolerability-and-pharmacodynamics-of-subcutaneously-administered-den-181-in-a/.

b https://anokion.com/wp-content/uploads/2019/09/ECTRIMS_Poster_9.13.19.pdf.

c MULTIPLE SCLEROSIS JOURNAL Volume: 25 Special Issue: SI Supplement: 2 Pages: 894−894 Published: SEP 2019.

d https://selectabio.com/immtor/gouttherapy/phase2results.

e Volume: 158 Issue: 6 Supplement: 1 Pages: S135-S135 Published: MAY 2020.

f https://clinicaltrials.gov/ct2/show/NCT03272269 (no results posted at time of review).

As reviewed elsewhere, the liver is especially important for tolerance induction [24]. The liver recycles cells and material from the rest of the body and, therefore, needs fail-safe mechanisms to maintain tolerance to self-antigens. A prime example is ageing or damaged red blood cells [25]: this recycling pathway is now being targeted for tolerance induction via liver APC [26]. Liver sinusoidal endothelial cells (LSECs) are highly effective in promoting TGF-β-dependent, Foxp3^+^ Treg when compared with other liver cell types [27]. Myelin peptides targeted to LSECs with iron oxide nanoparticles induced antigen-specific Treg that controlled EAE in mice [28^••^].

Nanoparticles and liposomes allow encapsulation of antigen or delivery with immunosuppressants and are believed to reduce the risk of immunogenicity of poorly soluble peptides and proteins. The fate of antigenic particles, from peptides to nanoparticles, depends on their size. Berkland *et al.* have shown that particles >200 nm are retained in the liver while those <4 nm are excreted [29]. Small particles, including soluble peptides, drain rapidly from sites of injection with particles of 4–10 nm penetrating the lymph node cortex to encounter ssDCs. Particles >100 nm, on the other hand, are retained in the subcapsular space where they are processed by macrophages. Nanoparticle approaches in clinical development range from medium sized particles enclosing rapamycin [30^••^] to larger particles taken up by monocytes that then traffic to lymphoid tissues and liver where they release antigen [31^••^]. Subcutaneous administration of liposomes encapsulating peptide antigen and calcitriol targets PD-L1^hi^ DCs, reduces their MHC class II expression, suppresses expansion of T effector cells and induces both antigen-specific Foxp3^+^ and IL-10^+^ Tregs [32^••^].

Recently, Krienke *et al.* described the use of RNA vaccines as a means of delivering autoantigenic peptides for AIT [33^••^]. Systemic delivery of 1-methylpseudouridine-modified mRNA coding for disease-associated T-cell epitopes resulted in their expression and antigen processing by CD11c^+^ ssDC, a reduction of effector T-cells and differentiation of Tregs capable of bystander suppression. It would be a major advantage if this approach could safely deliver whole antigens for AIT.

**Figure 2:**
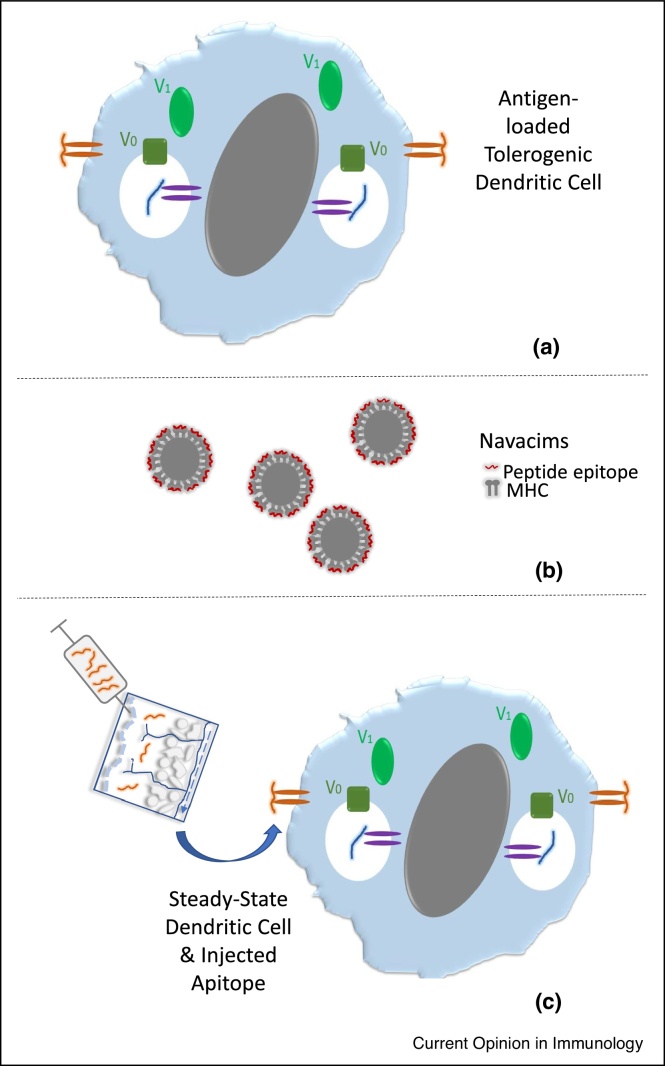
‘Direct’ delivery of antigenic epitopes for tolerance induction: **(a)** immature DCs generated *in* vitro and treated with drugs to block maturation are incubated with peptide antigens *in vitro*, the peptides bind to MHC and stabilise the complex. The resulting tolerogenic DC are injected by intravenous, intradermal or intranodal routes for direct presentation of these epitopes to T cells. **(b)** Navacims are nanoparticles coated with MHC-peptide complexes. In mice, repeated injection of Navacims (∼10x) induces antigen-specific Tr1 cells. **(c)** Apitopes, soluble T cell epitopes, are injected intradermally or subcutaneously. Peptides migrate rapidly to lymphoid organs via blood or lymph where they bind directly to and stabilise MHC II proteins on or in ssDC. Repeated injection of apitopes induces antigen-specific Treg and Tr1 cells to control autoimmune disease.

‘Direct’ antigen presentation for AIT has been achieved in three ways (Figure 2). First, human DC are generated *in* vitro, rendered tolerogenic by culture with vitamin D3, NFκB inhibitors or IL-10, combined with peptide epitopes and injected by intravenous, intradermal or intranodal routes. Phase I clinical trials have shown that this approach is well tolerated with preliminary evidence of efficacy [34^•^,35^•^,^36^].

Secondly, ‘direct’ exposure of T-cells to peptide epitopes may be achieved by loading them onto MHC-coupled nanoparticles (Navacims). Navacims have been shown to suppress a range of autoimmune disease models in mice [37^•^,38^••^]. They modify the function of previously activated Th1 cells rather than naïve T-cells converting them into cells with a Tr1 phenotype. The nanoparticles depend on accurate spacing of MHC molecules for optimal efficacy [39] and this could complicate large-scale manufacture.

Finally, peptides defined as apitopes (antigen processing independent T-cell epitopes) also bind directly to MHC II. Apitopes mimic naturally processed epitopes from self-antigens, suppress inflammatory cytokine secretion and promote differentiation of Tr1 cells, reviewed in Ref. [40]. Apitope mediated Tr1 cell differentiation is directly related to affinity for MHC with the resulting cells mediating negative feedback regulation by suppressing DC maturation. Repeated administration of apitopes drives a sequential change in gene expression [41^•^] with expression of tolerance genes similar to that seen in tumour infiltrating lymphocytes and other forms of induced tolerance [42^••^]. Analysis of cell signalling following tolerance induction showed that the resulting anergic, Tr1 cells have a membrane proximal block in signalling substantially limiting levels of downstream transcription factors [43^••^].

Recent work has shown that tolerance-associated genes induced in Tr1 cells, including IL-10, inhibitory receptors (CTLA-4, TIGIT, Tim-3 etc.) and IL-10-regulating transcription factors, are all modified by epigenetic priming [43^••^]. This opens chromatin close to tolerance-associated genes making them sensitive to low levels of transcription factors. This novel mechanism explains how potentially pathogenic T-cells transform into regulatory Tr1 cells mediating the negative feedback associated with apitope-induced AIT. This novel mechanism combining reduced TCR-mediated signalling with selective epigenetic changes may also account for the Th1-Tr1 conversion by navacims [37^•^] or the negative feedback regulation seen in chronic infections [44] and tumour infiltrating cells [42^••^].

## Apitopes selectively bind steady state DC

Apitopes administered by subcutaneous or intranasal delivery migrate rapidly to lymphoid organs and can be detected on splenic APC within minutes [45]. Recent work has revealed that apitopes bind rapidly and selectively to ssDC rather than B cells and monocytes following subcutaneous injection [46^•^]. Santambrogio *et al.* have shown that splenic ssDC have unstable MHC II at the cell surface that is stabilised by exogenous peptide whereas B cells and monocytes do not [47]. DC maturation is controlled by TLRs, cytokine receptors and costimulation [48] and correlates with both the loss of peptide-receptive MHC II at the cell surface [47] and upregulation of costimulatory molecules (Figure 1). Soluble apitopes, therefore, selectively target tolerogenic ssDC because these cells express unstable MHC II; but, why do ssDC have peptide-receptive MHC at the cell surface? There are various properties that distinguish tolerogenic ssDC from mature DC; however, the critical, distinguishing changes affecting antigen processing and presentation are governed by endosomal pH. Trombetta *et al.* have shown that the endosomal pH in immature DCs is high compared to mature DC [49] and this is governed by the 2 subunit V-ATPase. DC maturation induces the recruitment of cytosolic V-ATPase subunits to the endosomal/lysosomal membrane to create the ion pump required for acidification. Acidification is essential for the optimal function of pH-dependent proteases and chaperone proteins such as DM [50] in antigen processing and presentation. This explains why immature ssDC fail to process and present antigens effectively and why peptide-receptive MHC II appears at the cell surface. The low level of costimulatory molecules expressed by ssDC [48] renders them tolerogenic. This makes ssDC the ideal target for AIT since they remain tolerogenic if the peptide epitope is delivered without PAMPs or DAMPs (Figure 1).

## Clinical development of AIT with apitopes

The ultimate test of any therapeutic approach is its safe and effective application in the clinic. As yet, few of the approaches described above, other than tolerogenic DCs [34^•^,35^•^] or apitopes [18^••^,19^••^], have published outcomes of clinical trials (Table 1). In total, 78 patients have been treated with apitopes with no unexpected side effects. Furthermore, a phase I trial of apitopes in Graves’ disease, with epitopes from the TSHR [51^•^], noted a reduction in both thyroid hormone secretion and anti-TSHR antibodies in 7/10 patients [19^••^]. A phase 1b trial of intradermal apitopes from MBP in relapsing-MS [52^•^], reported a significant (*p* = 0.03) reduction in gadolinium-enhancing lesions while a phase 2 study revealed a marked improvement in cognition that correlated with suppression of CNS inflammation [18^••^]. The results of these trials show that the use of ssDC-targeting apitopes holds promise as a specific immunotherapy for a range of autoimmune conditions.

## Conclusions

In 2000, Harrison and Hafler stated that AIT for autoimmune diseases was ‘closer to the Holy Grail’ [53]. The intervening years have seen evolution of novel approaches to target self-antigens to APCs including ssDC and liver APC. In our view, there are three questions that must be addressed by each approach. What is the mechanism of action in affected individuals and does this allow their safe administration to individuals? Does the approach induce bystander suppression? Most autoimmune diseases involve more than one antigen: a bystander-suppressive mechanism will be needed to control epitope spreading [54]. Does the approach allow repeated administration? Our experience of AIT with apitopes shows that repeated exposure will be required for maintenance of tolerance. Continuing progress in this field convinces us that AIT for autoimmune diseases is now ‘reaching the Holy Grail’.

## Conflict of interest statement

HS and DW declare stock ownership in Apitope International NV. DW serves as a consultant to Apitope International NV and has sat on scientific advisory boards for Actelion Pharma and Zealand Pharma; is a senior editor for Immunotherapy; holds patents for peptides, tolerisation-inducing composition, FVIII peptides and their use in tolerising haemophiliacs, composition, disease markers, tolerogenic peptides from myelin basic protein, peptide selection method, and improvements relating to influenza vaccine; has consulted for Peptide Therapeutics Ltd., Teva, GSK Bio, Hoffmann-La Roche, Novartis, DTI, and the Food Standards Agency; received research support within the past 3 years from UCB Celltech and was an expert witness for Geron.

## References and recommended reading

Papers of particular interest, published within the period of review, have been highlighted as:

• of special interest
•• of outstanding interest
